# Pink–White Esthetic Optimization in Excessive Gingival Display Using Crown Lengthening and Direct Composite Veneering

**DOI:** 10.1155/crid/9631149

**Published:** 2026-05-28

**Authors:** Nihangma Patangwa, Sanjay Kumar Sah

**Affiliations:** ^1^ Prosthodontics Unit, Department of Dental Surgery, Bir Hospital, National Academy of Medical Sciences, Kathmandu, Nepal, nams.org.np

**Keywords:** dental proportion, esthetic rehabilitation, minimally invasive restoration, surgical crown lengthening

## Abstract

An esthetic smile plays a pivotal role in enhancing patient confidence and social well‐being. Smile esthetics are influenced by multiple factors, including maxillary anterior tooth morphology, lip length and mobility, periodontal health, and the degree of gingival display. Short clinical crowns associated with excessive gingival display are generally perceived as unesthetic and frequently require esthetic crown lengthening. Altered active eruption (AAE) and altered passive eruption (APE) represent common etiologic factors in such presentations. Management typically involves gingivectomy with or without osseous resection while ensuring adequate space for the supracrestal tissue attachment. Violation of the supracrestal tissue attachment may lead to gingival inflammation, recession, and alveolar bone loss; therefore, a postoperative healing period of 3–6 months is recommended to allow soft tissue maturation and assessment of marginal gingival stability. Conventionally, restorative rehabilitation following esthetic crown lengthening has relied on invasive approaches, including porcelain‐fused‐to‐metal crowns, all‐ceramic crowns, and porcelain veneers. However, evidence supporting minimally invasive or noninvasive restorative modalities—particularly direct composite restorations—remains limited. This case series is aimed at describing a minimally invasive restorative protocol utilizing direct nanocomposite veneering following esthetic crown lengthening and soft tissue maturation to achieve favorable pink esthetic score (PES) and white esthetic score (WES) outcomes. Two patients presenting with a gummy smile, microdontia, and spacing in the maxillary anterior region were diagnosed with APE Type I associated with AAE and exhibited low baseline PES and WES values. Diagnosis was established through clinical transgingival probing, cone‐beam computed tomography (CBCT), diagnostic casts, and digital smile design. Treatment involved esthetic crown lengthening via gingivectomy and ostectomy, followed by a 3‐month healing period for soft tissue maturation. Subsequently, minimally invasive direct veneering was performed using a bioactive nano‐hybrid composite. This approach resulted in improved pink and white esthetic outcomes while preserving tooth structure and maintaining periodontal health. At approximately 24 months of follow‐up, minor restoration chipping was observed, along with mild gingival inflammation associated with plaque accumulation, whereas gingival levels remained relatively stable, ranging from −1.5 to +0.5. The primary limitation of this case series is the absence of long‐term data regarding color stability, periodontal response and restorative complications.

## 1. Introduction

An esthetic smile contributes significantly to patient confidence and psychosocial well‐being [1–3]. Several guidelines for smile esthetics have been proposed, notably by Lombardi et al. and Magne et al. [4, 5]. An ideal smile is characterized by approximately 1 mm of gingival display between the gingival margin and the upper lip, with complete exposure of the clinical crowns of the maxillary anterior teeth [6]. The maxillary anterior dentition plays a dominant role in smile esthetics; however, additional contributing factors include lip length, lip mobility, periodontal health, and the extent of gingival display [7].

Short clinical crowns accompanied by excessive gingival display are generally considered unesthetic and often necessitate esthetic crown lengthening [8–11]. Indications for esthetic crown lengthening include altered passive eruption (APE), altered active eruption (AAE), subgingival caries, tooth or root fractures, and cervical root resorption [8, 9]. Active eruption refers to the occlusal movement of teeth, whereas passive eruption involves the apical migration of the gingival margin to expose the anatomical crown [12, 13]. AAE occurs when the distance between the cementoenamel junction (CEJ) and the alveolar bone crest is less than 1.5–2 mm, often due to insufficient occlusal movement during eruption [10]. In contrast, APE is characterized by a gingival margin that remains coronally positioned after tooth eruption, resulting in coverage of the cervical portion of the crown [10, 14].

Coslet et al. proposed a classification system for APE based on the amount of keratinized gingiva and the position of the alveolar bone crest relative to the CEJ [14]. Type I is characterized by excessive gingival tissue and a wide band of keratinized gingiva, with the mucogingival junction positioned apical to the alveolar bone crest, whereas Type II presents with a narrow band of keratinized gingiva and the mucogingival junction located at the level of the CEJ. Both types are further subdivided into Subtype A, in which the bone crest lies 1.5–2 mm apical to the CEJ, allowing adequate connective tissue attachment, and Subtype B, in which the bone crest is at or near the CEJ, compromising the supracrestal tissue attachment [14]. Subsequently, Ragghianti proposed a modified classification that retained Types I and II based on the width of keratinized gingiva (> 2 mm and < 2 mm, respectively) and introduced Subgroup A (APE alone) and Subgroup B (APE associated with AAE) to facilitate diagnosis [10].

Esthetic crown lengthening accounts for approximately 10% of all periodontal surgical procedures, as reported by the American Academy of Periodontology [15]. A critical principle underlying the crown lengthening procedure is the preservation of the supracrestal tissue attachment, defined as the combined dimension of the junctional epithelium and connective tissue attachment extending from the base of the gingival sulcus to the alveolar bone crest [16]. Gargiulo et al. reported an average supracrestal tissue attachment of 2.04 mm [17]. However, a systematic review has demonstrated considerable variability, with reported values ranging from 0.2 to 6.73 mm [18]. Violation of the supracrestal tissue attachment has been associated with adverse periodontal sequelae, including inflammation, gingival recession, and alveolar bone loss [19–21].

Restorative intervention is integral to achieving optimal smile esthetics. To date, the majority of published literature describes invasive restorative protocols involving tooth preparation followed by placement of porcelain‐fused‐to‐metal or all‐ceramic crowns [22, 23]. Minimally invasive approaches using lithium disilicate restorations and porcelain veneers have been reported in isolated case studies [24]. Indirect ceramic veneers offer advantages of superior color stability, exceptional marginal integrity, biocompatibility with gingival tissue, and long‐term fracture resistance [25]. Mazzetti et al. in a 10‐year practical‐based evaluation of ceramic and direct composite veneers found that ceramic veneers had superior longevity than direct composite veneers in both success and survival analysis [26]. Meanwhile, Korkut and Türkmen et al. [27] found that the survival rate of direct diastema closure and recontouring of direct composite veneers in a 4‐year clinical trial was 90% [27]. Direct composite veneers are often preferred when preservation of tooth structure is a priority. It is usually the choice of treatment for adolescents and young adults with intact enamel surfaces [28, 29]. Although direct composite exhibits disadvantages like chipping, surface roughness, wear, and marginal discoloration, it can be easily repaired cost‐effectively simply by adding composite and polishing, avoiding the more catastrophic complications such as fracture in terms of indirect ceramic veneers [30, 31]. A systematic review and meta‐analysis concluded that the dental veneers, both direct composite and indirect ceramic veneers, have a positive impact on overall periodontal health in individuals [32]. This finding is consistent with the result of a retrospective study that even deep subgingival direct composite restorations were not associated with increased periodontal or gingival response over a period of 3 years [33]. However, evidence supporting the use of direct composite resin restorations for improving white esthetics following esthetic crown lengthening remains scarce. Therefore, the purpose of this case series is to describe a noninvasive restorative protocol employing direct composite veneering after esthetic crown lengthening to achieve satisfactory pink and white esthetic outcomes.

## 2. Case Description

### 2.1. Inclusion Criteria

The inclusion criteria were designed to identify patients who required a combined periodontal‐restorative approach to optimize both pink and white esthetics. The two cases were selected based on the following clearly defined parameters:

• Clinical diagnosis of excessive gingival display, where both patients presented with a gummy smile characterized by 3–4 mm (Case 1) and 5–6 mm (Case 2) of gingival exposure during smiling.
• Definitive diagnosis of APE Type I with AAE established through clinical transgingival probing and cone‐beam computed tomography (CBCT), which confirmed that the alveolar bone crest was at or near the level of the CEJ.
• Restorative indications (white esthetics) where both patients exhibited generalized microdontia and maxillary anterior spacing, which necessitated restorative intervention following surgical crown lengthening to achieve ideal tooth proportions.
• Patients with a thick gingival phenotype and an adequate width of keratinized gingiva were included to allow for predictable gingivectomy and phenotype modification while maintaining periodontal stability [34].

### 2.2. Case 1

A 24‐year‐old female presented to the Prosthodontics Unit, Department of Dental Surgery, NAMS, with a chief complaint of small maxillary anterior teeth with spacing. She was primarily concerned about esthetics and had lost confidence due to an unpleasant smile. Her medical history was noncontributory. Dental history revealed that she had undergone orthodontic treatment and had recently had her braces removed.

**Figure 1:**
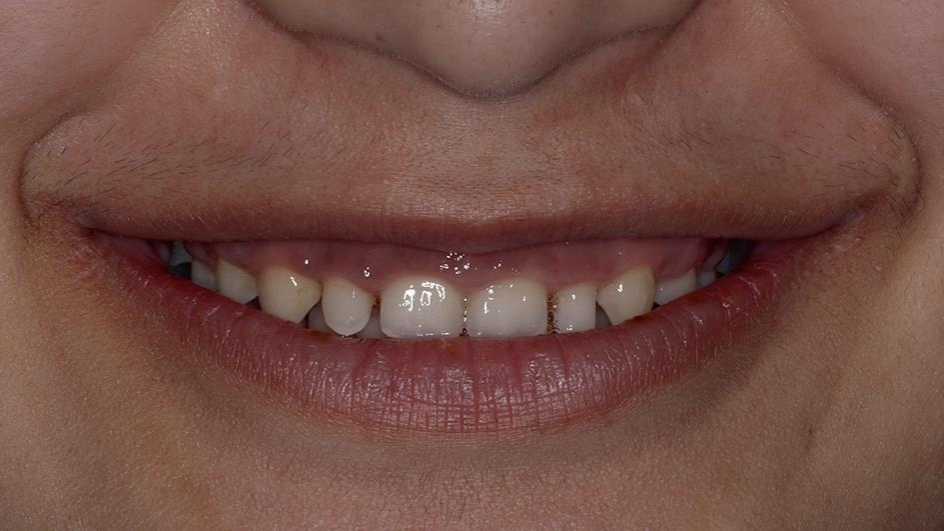
Gummy smile with microdontia and spacing.

On extraoral examination, she presented with an apparently symmetrical face and lips with normal mobility. The maxillary anterior teeth were clinically short in both length and width, with spacing present between the maxillary right second premolar to left second premolar (Table 1). On smiling, she exhibited a gummy smile with approximately 3–4 mm of gingival exposure. The dental midline did not coincide with the facial midline (Figure 1).

**Table 1:** Preoperative clinical crown height (mm) measured from the gingival zenith to the incisal edge for Teeth 15–25 in Cases 1 and 2.

Tooth number	Case 1: Preoperative clinical crown height (mm)	Case 2: Preoperative clinical crown height (mm)
15	3.09	5.26
14	4.88	5.43
13	5.35	4.98
12	4.83	5.22
11	5.70	6.27
21	5.87	6.25
22	4.46	5.76
23	5.54	5.08
24	4.68	4.59
25	3.58	4.20

**Figure 2:**
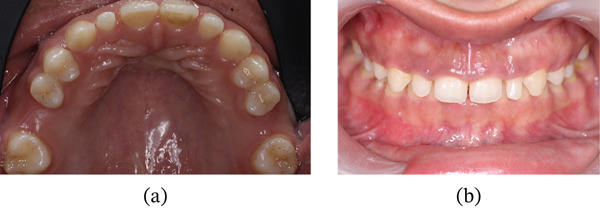
(a) Maxillary arch with spacing and missing 16 and 26. (b) Increased vertical overlap.

**Figure 3:**
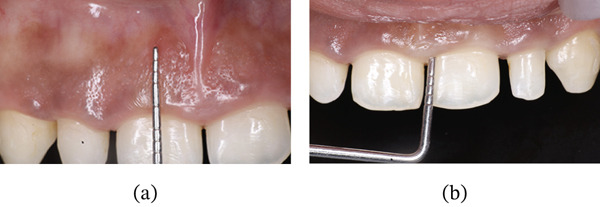
(a) Width of attached gingiva. (b) Measurement of probing depth.

On intraoral examination, Teeth 16 and 26 were missing, with inadequate space remaining. Increased vertical overlap was present, with complete coverage of the mandibular anterior teeth. The mandibular anterior teeth were also clinically short and retained with fixed retainers; however, the patient did not desire treatment in the mandibular arch (Figure 2a,b). Periodontal examination of the maxillary anterior region revealed a thick gingival biotype, with a width of keratinized gingiva measuring 6–7 mm and probing depths ranging from 5 to 6 mm (Figure 3a,b) [34].

**Figure 4:**
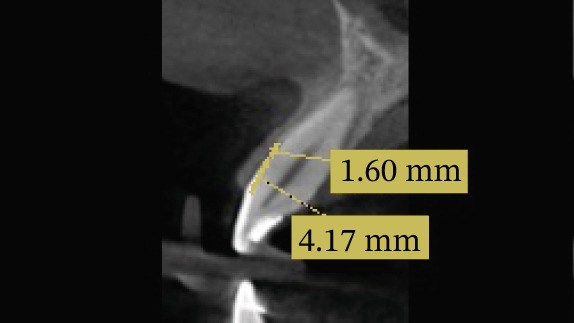
The distance between CEJ and the alveolar crest is 1.60, and the marginal gingiva is 4.17 mm above the CEJ.

**Figure 5:**
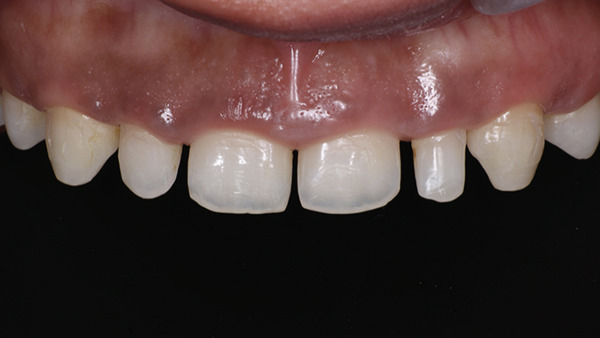
Evaluation of white and pink esthetic score.

CBCT was prescribed for radiographic evaluation following the initial examination. The distance between the CEJ and the alveolar bone crest ranged from 1.3 to 4.36 mm (Figure 4 and Table 2). Based on these findings, a diagnosis of APE Type I with AAE was established, with pink esthetic score (PES) and white esthetic score (WES) values of 6 and 7, respectively [10, 35] (Figure 5). A treatment plan consisting of esthetic crown lengthening from right second premolar to the left second premolar, followed by direct composite veneering of Teeth 13, 12, 11, 21, 22, and 23, was proposed and approved by the patient. Implant placement was planned for teeth 16 and 26.

**Table 2:** Bone–cementoenamel junction (CEJ) distance measurements (mm) from Teeth 15–25.

Tooth number	Case 1: Bone–CEJ distance (mm)	Case 2: Bone–CEJ distance (mm)
15	2.23	1.40
14	2.54	1.91
13	2.13	1.90
12	1.35	1.98
11	1.60	2.65
21	1.62	1.96
22	2.95	2.26
23	4.36	2.10
24	3.34	2.13
25	3.38	2.13

**Figure 6:**
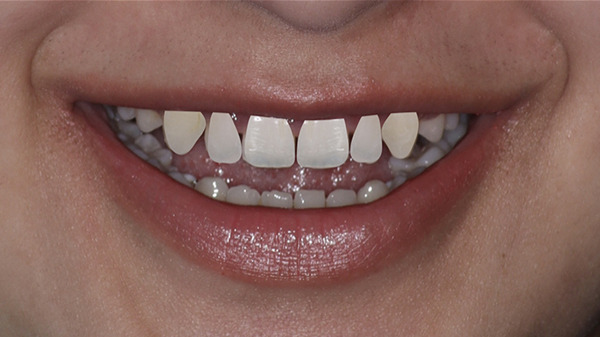
Digital smile design.

Extraoral and intraoral photographs were obtained. A diagnostic impression was made using alginate, and a cast was poured. The length and width of five teeth in each maxillary quadrant were measured on the cast and used for smile design. Digital smile design (DSD) was performed using Keynote software, and the proposed future clinical crown lengths were derived for reference (Figure 6). Following patient approval of the esthetic outcome, a surgical appointment was scheduled. The patient was informed about the treatment plan and the prognosis, and an informed consent was obtained before starting the procedure.

**Figure 7:**
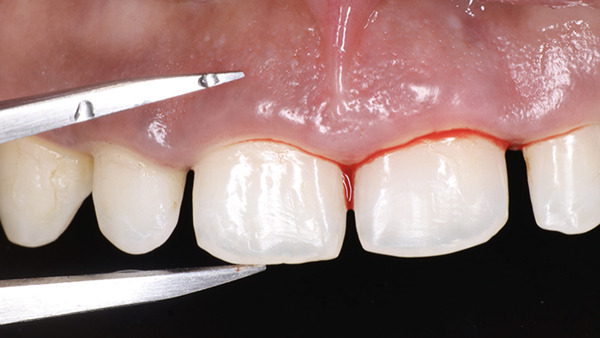
Future gingival zenith marked with the reference length acquired from DSD.

**Figure 8:**
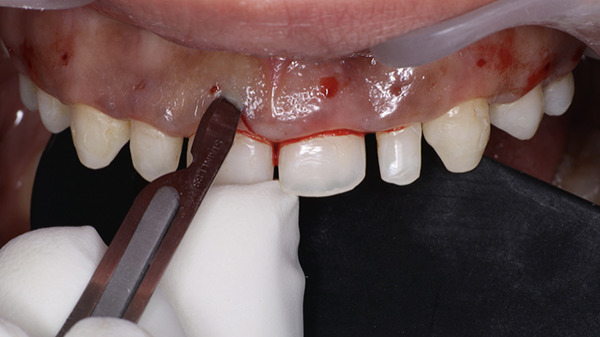
Internal bevel incision.

**Figure 9:**
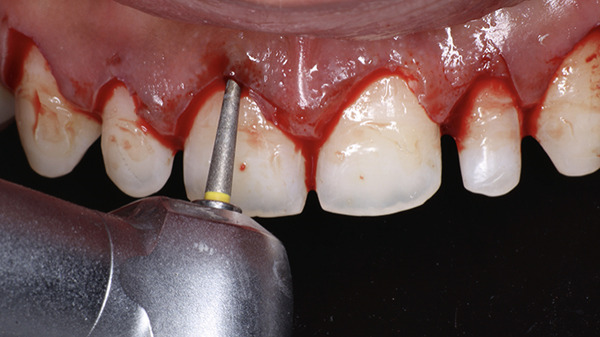
Gingival thinning with a flame‐shaped diamond.

At the surgical appointment, local anesthesia was administered using lignocaine hydrochloride with epinephrine (1:80,000). Based on measurements obtained from the DSD, the future position of the marginal gingiva was marked on each tooth using a Castroviejo caliper (Figure 7). Markings were placed distal to the midline for both central and lateral incisors and at the midline for canines and premolars. An internal bevel incision was made using a 15C blade (Hu‐Friedy) following the natural tooth contour (Figure 8). The flatter and thinner 15C blade with a more acute taper and an extended cutting edge, when compared with a traditional 15 blade, provides superior precision when performing delicate work along the scalloped anatomy of the CEJ. Subsequently, an intrasulcular incision was performed, and the gingival collar was removed using a periodontal curette (Hu‐Friedy). Gingival thinning was carried out using a flame‐shaped superfine diamond bur (Shofu Dental SF215) for gingival phenotype modification (Figure 9).

**Figure 10:**
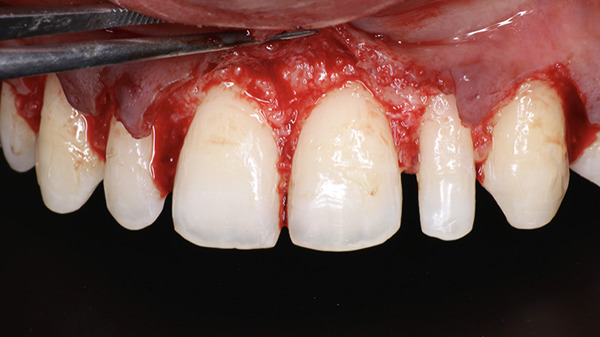
Full‐thickness flap with CEJ coinciding with the alveolar crest.

**Figure 11:**
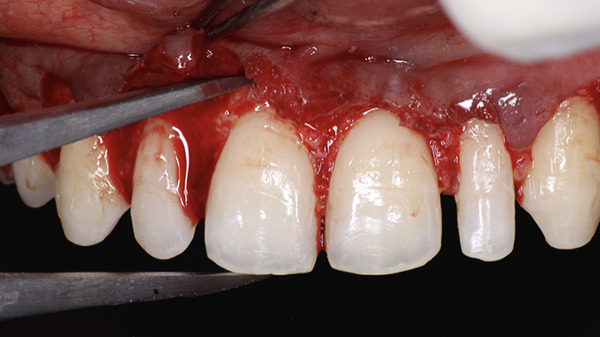
3 mm added to allow space for supracrestal tissue attachment.

**Figure 12:**
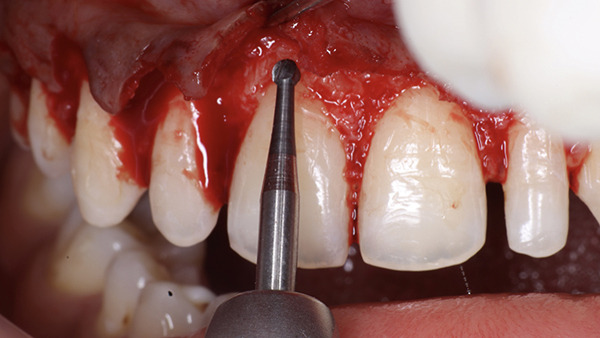
Ostectomy done with a round carbide bur in a slow‐speed handpiece.

**Figure 13:**
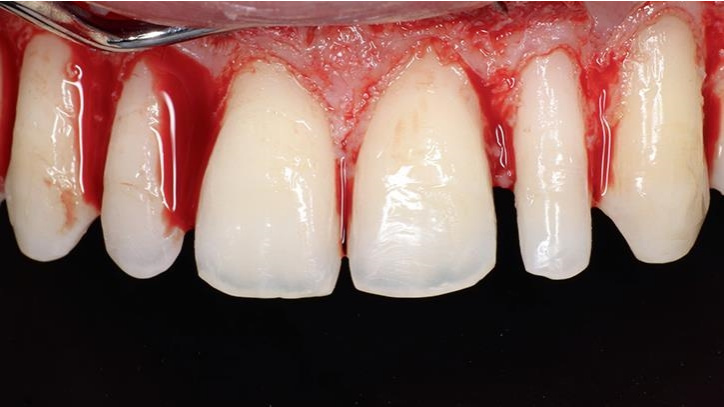
Postostectomy.

**Figure 14:**
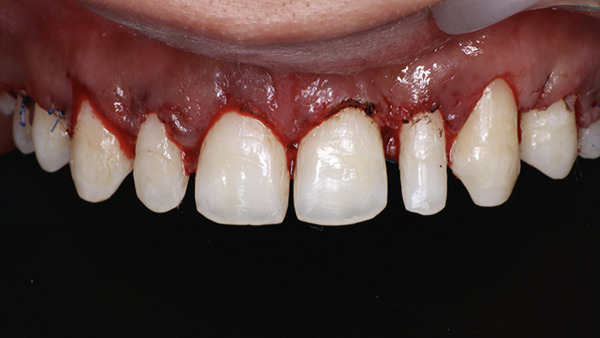
Vertical mattress suturing of the full‐thickness flap.

A full‐thickness labial and buccal flap was elevated coronal to the mucogingival junction. The alveolar bone crest was found to be at the level of the CEJ (Figure 10). Three millimeter was added using the Castroviejo caliper and marked to allow adequate space for the supracrestal tissue attachment (Figure 11). Osteotomy was performed using a carbide round bur (Omega surgical instruments, 5‐mm diameter) in a slow‐speed straight handpiece, following the contour of the CEJ (Figure 12). Vertical grooving and festooning were completed, ensuring that no steps or ledges remained at the junction of the alveolar bone crest and root surface (Figure 13). The surgical site was irrigated with saline solution, and the flap was repositioned. Digital pressure was applied for 1 min, and the flap was stabilized using 6‐0 polypropylene sutures placed in a vertical mattress configuration (Figure 14).

**Figure 15:**
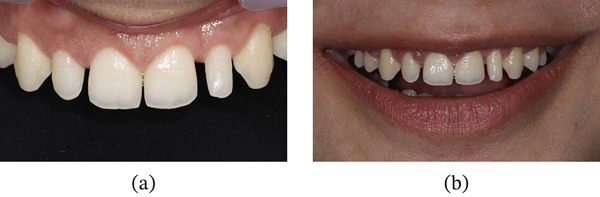
Soft tissue maturation after 3 months postoperatively. (a) Intraoral view. (b) Smile view.

Postoperative instructions were provided, and medications including paracetamol with ibuprofen and amoxicillin 500 mg three times daily were prescribed for 5 days. A 0.12% chlorhexidine mouthwash was advised twice daily for 2 weeks. The patient was instructed to refrain from brushing the surgical area for 2 weeks and to apply ice packs during the first 24 h. Additionally, she was advised to consume a soft diet during the first postoperative week and to avoid mechanical trauma. Sutures were removed 10 days postoperatively, and the soft tissues were allowed to mature for 3 months (Figure 15a,b).

**Figure 16:**
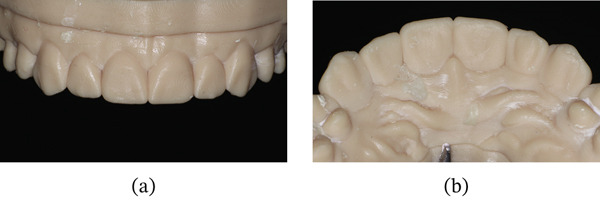
3D model of a digital wax‐up. (a) Frontal view. (b) Palatal view.

A digital impression was then made for diagnostic wax‐up. After confirmation of the digital wax‐up, a three‐dimensional model was printed (Figure 16a,b). A palatal index was fabricated using addition silicone.

**Figure 17:**
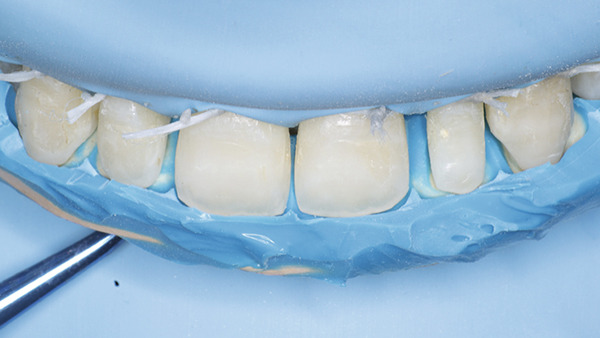
Rubber dam placed and floss tied along with the palatal index.

**Figure 18:**
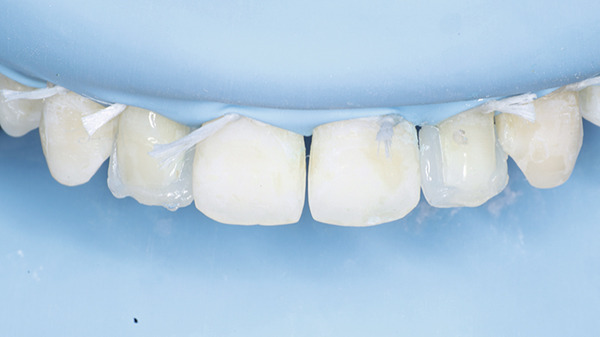
Bilaminar natural layering/shading technique.

At the restorative appointment, rubber dam isolation was achieved, and dental floss ligatures were secured from Teeth 15 to 25 to maintain isolation and retraction [36]. The teeth were polished with pumice slurry, and the fit of the palatal index was verified (Figure 17). The enamel surfaces were etched with 37% phosphoric acid for 30 s, followed by thorough rinsing and drying. A bonding agent was applied, gently air‐thinned to maintain a uniform layer, and light‐cured for 20 s. A bilaminar natural layering technique was employed for direct veneering [37]. Using the palatal index, a palatal shell was constructed with enamel composite (Beautifil II Enamel), and proximal walls were built using sectional matrices (Tor VM sectional contoured metal matrices—Universal kit) and enamel composite (Figure 18).

**Figure 19:**
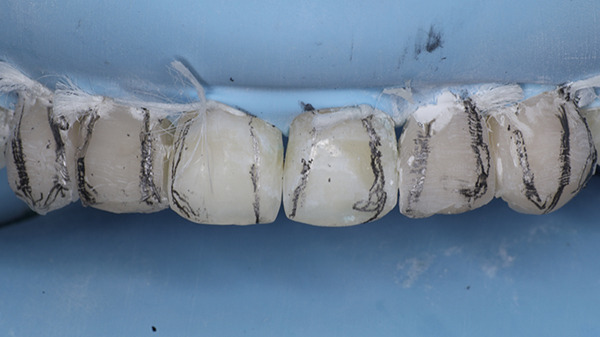
Marking of transitional lines with a pencil.

**Figure 20:**
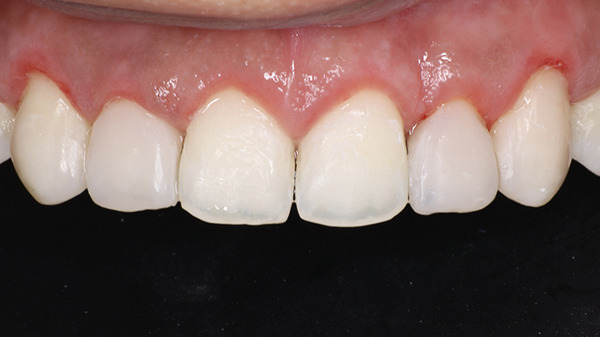
After 24 h of completion of the restorative phase.

A bioactive nano‐hybrid composite (Beautifil Injectable XSL, Shade A2) was injected, sculpted using a probe, and light‐cured for 20 s. A final enamel layer was applied to complete the restorations and light‐cured for an additional 20 s. Excess restorative material was carefully removed using a No. 12D surgical blade (Hu‐Friedy). Transitional line angles were marked with a pencil, and a fine‐grit flame‐shaped diamond bur (Shofu Dental F216) was used to define the primary anatomy (Figure 19). Following rubber dam removal, gross finishing was performed using Super‐Snap discs (Shofu dental) at slow speed. Proximal contours were refined using interproximal finishing strips (Shofu Super‐Snap Polystrips) with an S‐shaped technique. Final polishing was carried out using Diacomp EVE spirals (pink followed by gray) with minimal pressure and intermittent strokes. The patient was recalled after 24 h for final finishing and polishing (Figure 20).

**Figure 21:**
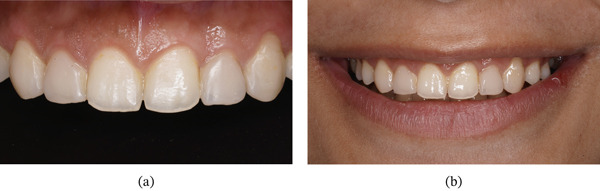
Six months of follow‐up. (a) Frontal view. (b) Smile view.

**Figure 22:**
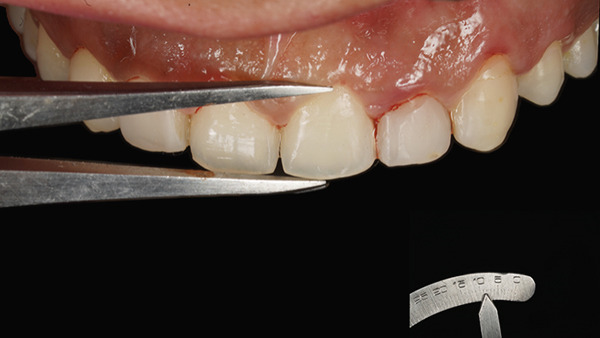
Twenty‐four months of follow‐up frontal view shows stable clinical crown lengths.

Following completion of the restorations, the PES and WES values improved to 9 and 9, respectively. At the 6‐month follow‐up, the periodontal tissues were healthy with no evidence of gingival relapse, and the restorations remained intact (Figure 21a,b). At the 24‐month follow‐up, minor chipping of the composite restoration with respect to 22 was noted and was subsequently repaired. Mild gingival inflammation was observed and minimal gingival rebound (coronal migration of the margin) ranging from 0 to 0.5 mm, suggesting that the surgical protocol effectively established and maintained the desired crown dimensions over time (Figure 22 and Table 3).

**Table 3:** Gingival rebound quantification—Case 1.

Tooth number	Preoperative crown height (mm)	Immediately After crown lengthening (mm)	3‐month postoperative crown height (mm)	24‐months postoperative crown height (mm)	Gingival rebound (mm)
15	3.09	5.5	5.5	5.5	0
14	4.88	6.5	6.5	6.5	0
13	5.35	8.5	8.5	8	0.5
12	4.83	7	7	6.5	0.5
11	5.70	9	9	8.5	0.5
21	5.87	9.5	9.5	9	0.5
22	4.46	6.5	6.5	6.25	0.25
23	5.54	9.5	9.5	9	0.5
24	4.68	6.5	6.5	6.5	0
25	3.58	6	6	6	0

### 2.3. Case 2

**Figure 23:**
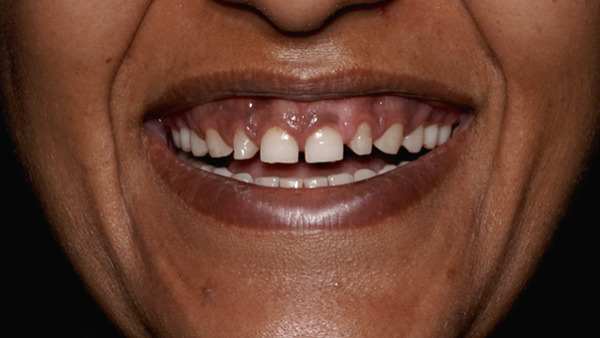
Gummy smile.

A 30‐year‐old patient visited the Prosthodontics Unit, Department of Dental Surgery, with a chief complaint of small teeth with spacing. The patient was mainly concerned about esthetics. She was apparently healthy, with no systemic diseases. On extraoral examination, the face was apparently symmetrical, with the facial midline coinciding with the dental midline. She had a high smile line with exposure of 5–6 mm of gingiva (Figure 23).

**Figure 24:**
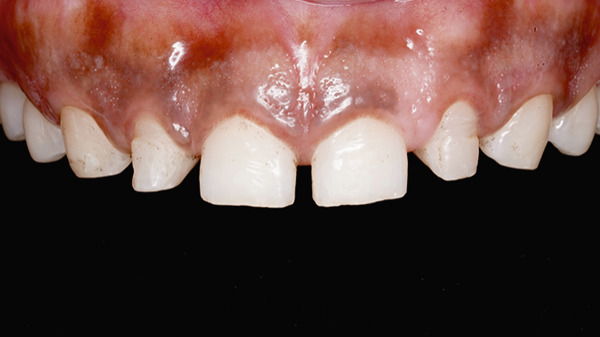
Microdontia, along with spacing with respect to 13, 12, 11, 21, 22, 23, and evaluation of pink and white esthetics.

**Figure 25:**
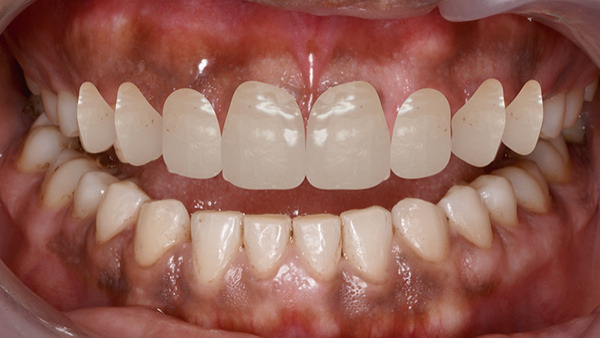
Digital smile design.

On intraoral examination, generalized microdontia with spacing with respect to Teeth 13, 12, 11, 21, 22, and 23 was present. The PES and WES scores of the patient were 5 and 4, respectively (Figure 24). Extraoral and intraoral photographs were taken. CBCT evaluation was performed for radiographic assessment. It was found that the alveolar crest was close to the CEJ with respect to Teeth 15, 14, 13, 12, 11, 21, 22, 23, 24, and 25 (Table 2). The length and width of each maxillary anterior tooth were measured intraorally with the vernier caliper and used as reference parameters for smile design (Table 1). DSD was performed using keynote software, through which the proposed future clinical crown lengths were determined to guide the treatment planning process. After DSD, the treatment plan of esthetic crown lengthening with ostectomy followed by direct composite veneering was confirmed with the patient (Figure 25). The patient was informed about the treatment plan and the prognosis, and informed consent was obtained before starting the procedure.

**Figure 26:**
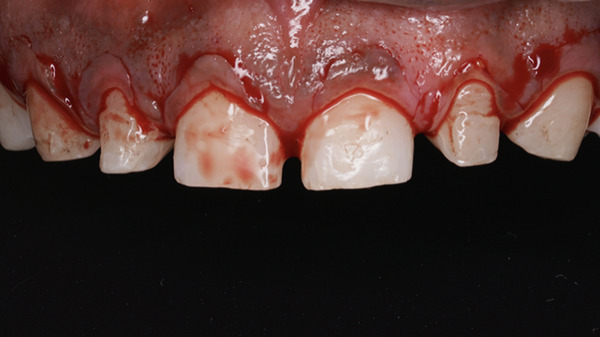
Internal bevel incision and intrasulcular incision given.

**Figure 27:**
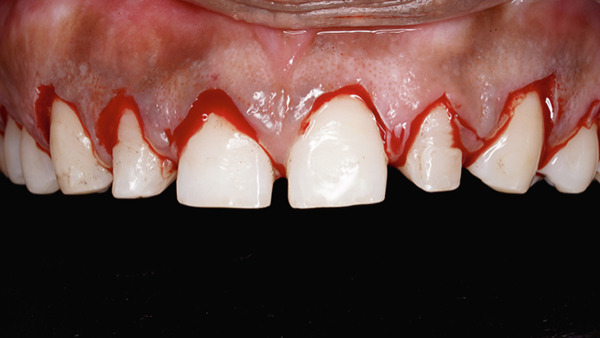
Removal of resected gingiva.

**Figure 28:**
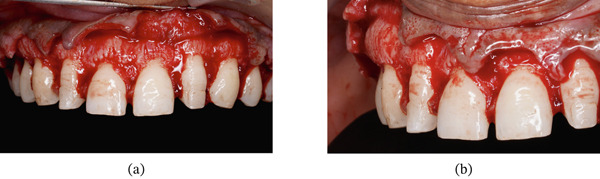
Buttressed bone formation. (a) Frontal view. (b) Lateral view.

During the surgical appointment, lignocaine with epinephrine (1:200,000) was administered as local anesthesia. A Castroviejo caliper was used to mark the amount of gingiva to be removed. A reference point 3 mm above the gingival zenith of the central incisors was marked, and using this as a reference, markings were subsequently made on the lateral incisors, canines, and premolars. Internal bevel incisions followed by intrasulcular incisions were performed using a 15C blade (Hu‐Friedy) (Figure 26). The resected gingiva was removed (Figure 27). Gingival thinning was performed using a flame‐shaped bur (Shofu Dental SF215). A full‐thickness mucoperiosteal flap was then raised coronal to the mucogingival junction. On flap elevation, buttressed bone formation was observed, especially in the canine and premolar regions (Figure 28a,b).

**Figure 29:**
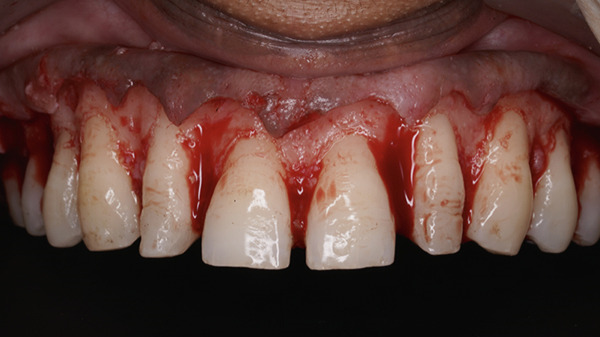
After ostectomy.

**Figure 30:**
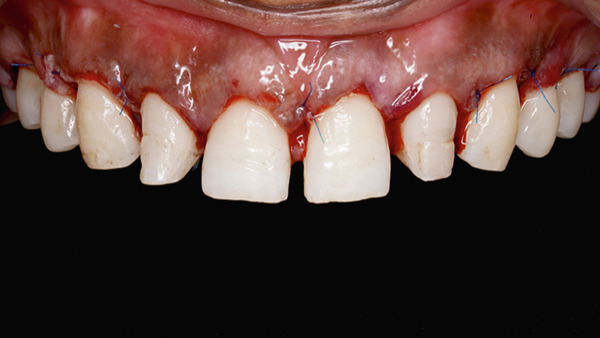
Vertical mattress suturing.

Using a Castroviejo caliper, 3 mm was marked apical to the CEJ to allow space for the supracrestal tissue attachment. Osteotomy of the buttressed bone and ostectomy extending 3 mm apical to the CEJ were performed using a round diamond bur (Omega surgical instruments, 5‐mm diameter) in a slow‐speed straight handpiece, followed by vertical grooving and festooning (Figure 29). Vertical mattress suturing of the flap was performed (Figure 30).

**Figure 31:**
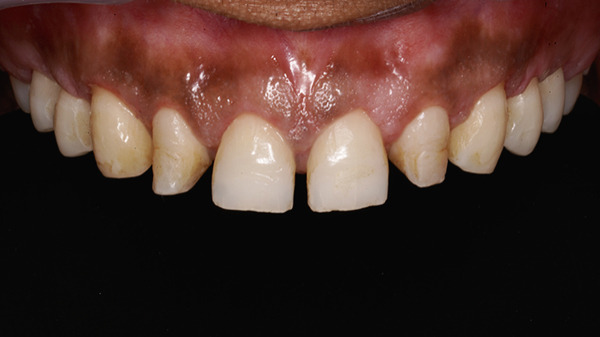
Three months follow‐up.

**Figure 32:**
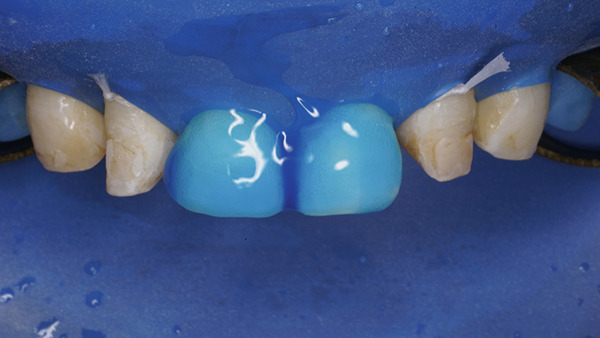
Rubber dam application, floss tie, and etching.

**Figure 33:**
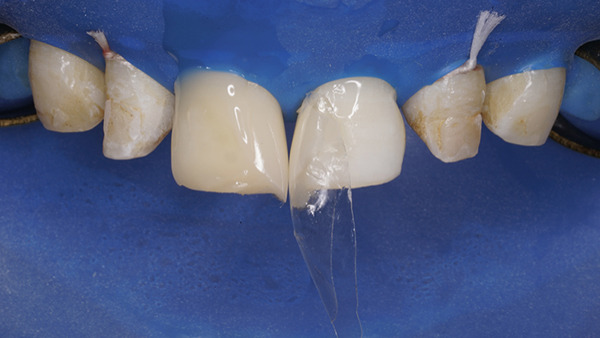
Use of bioclear matrix and nanocomposite for direct veneering.

**Figure 34:**
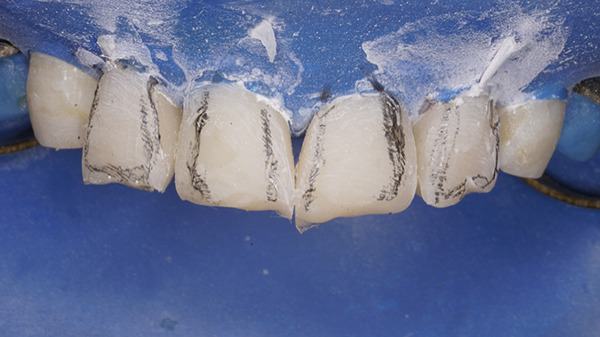
Marking line angles.

**Figure 35:**
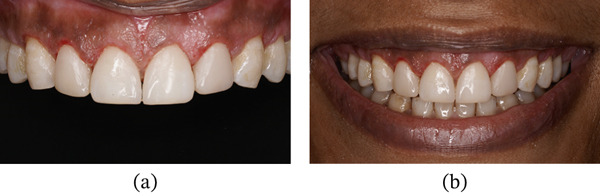
After finishing and polishing. Evaluation of pink and white esthetic score. (a) Frontal view. (b) Smile view.

**Figure 36:**
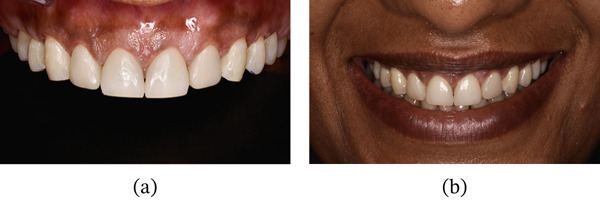
Six months follow‐up. (a) Frontal view. (b) Smile view.

**Figure 37:**
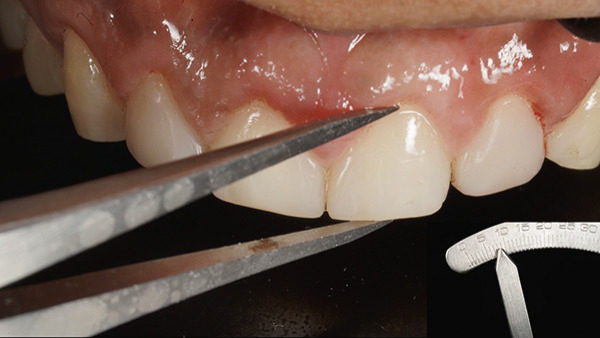
Twenty months follow‐up with stable clinical crown lengths.

After 3 months of soft tissue maturation, the patient was recalled for the restorative procedure (Figure 31). A rubber dam was placed from Teeth 15 to 25, and dental floss ligatures were secured. Etching was performed using 37% phosphoric acid (Figure 32), followed by application of a bonding agent. A Bioclear matrix was used to build the proximal walls and contact areas of the anterior teeth (Figure 33). A nanocomposite (Beautifil, Shofu) was used as the restorative material. After composite buildup, mesial and distal line angles were marked, and finishing was performed using flame‐shaped burs (Shofu dental, F216) and discs (Figure 34). Diacomp EVE was used for polishing the composite restorations. The postrestorative PES and WES scores were 10 and 9, respectively (Figure 35a,b). At the 6‐month follow‐up, the periodontal tissues were healthy with no evidence of gingival relapse, and the restorations remained intact (Figure 36a,b). At 20 months postoperative evaluation, the gingival margin remained remarkably stable with gingival changes ranging from −1.5 to 0 mm, along with mild inflammation (Figure 37 and Table 4). The apical migration of gingiva with respect to Tooth #14 might be attributed to possible recession.

**Table 4:** Gingival rebound quantification—Case 2.

Tooth number	Preoperative crown height (mm)	Immediately After crown lengthening (mm)	3‐month postoperative crown height (mm)	20‐months postoperative crown height (mm)	Gingival rebound (mm)
15	5.26	5.5	5.5	6	−0.5
14	5.43	6	6	7.5	−1.5
13	4.98	8.5	8.5	8.5	0
12	5.22	7.5	8	8	−0.5
11	6.27	9	9	9	0
21	6.25	9	9	9	0
22	5.76	7.5	7.5	7.5	0
23	5.08	8	8	8	0
24	4.59	7	7	7	0
25	4.20	6	6	6	0

## 3. Discussion

This case series evaluated the esthetic and periodontal outcomes of a minimally invasive treatment protocol combining esthetic crown lengthening with direct nanocomposite veneering in patients presenting with excessive gingival display and spacing in the maxillary anterior region. Both patients were diagnosed with APE Type I associated with AAE, accompanied by low baseline PES and WES values. The proposed approach aimed to restore harmonious pink and white esthetics while preserving tooth structure and maintaining periodontal health.

Accurate diagnosis was achieved through a comprehensive assessment that included clinical transgingival probing, CBCT, diagnostic casts, and DSD. In this case series, gingivectomy was performed using reference markings placed at the level of the gingival zenith, followed by ostectomy, which was also marked accordingly. Both markings were established using a Castroviejo caliper, and the gingivectomy and ostectomy procedures were carried out freehand. However, with recent advancements, the use of a 3D‐printed surgical guide for both gingivectomy and ostectomy is possible by integrating DICOM data with STL files obtained from an intraoral scanning (IOS) or from a digital wax‐up alone [38, 39]. Although the use of a surgical guide may reduce operating time and minimize the likelihood of measurement‐related human error, studies have shown no significant differences in wound healing, postoperative pain scores, or gingival margin stability when comparing conventional and computer‐guided esthetic crown lengthening procedures [38]. Both cases demonstrated a thick gingival phenotype with an adequate width of keratinized gingiva, allowing for predictable gingivectomy without compromising periodontal stability [40]. Maintenance of a minimum of 3 mm between the CEJ and the alveolar bone crest was emphasized to accommodate the supracrestal tissue attachment and minimize the risk of soft tissue rebound. Gingival phenotype modification through thinning further contributed to marginal gingival stability during the healing phase.

A soft tissue maturation period of 3 months was allowed, as the literature generally recommends a healing interval of 3–6 months to minimize soft tissue rebound [41, 42]. This selection is supported by Abdullah et al., who observed that although significant shifts occur within the first month, marginal gingival levels show no statistically significant difference between 3 and 9 months postsurgery [43, 44]. These results align with research by Lanning et al., indicating that the gingival margin achieves stability between the third and sixth postoperative months [45]. Therefore, clinicians should respect the need of periodontal tissues for an adequate healing time of ≥ 3 months prior to placement of definitive restoration [41]. However, positional stability is also heavily influenced by the periodontal phenotype, with thick phenotypes demonstrating significantly greater coronal soft tissue regrowth compared with thin phenotypes [41, 42]. This necessitates maintenance of approximately 3 and 2 mm between the alveolar crest and the CEJ for thick and thin phenotypes, respectively, to accommodate the reformed supracrestal tissue attachment [41, 42, 45]. Furthermore, Arora et al. reported that suturing the flap 3 mm from the alveolar crest resulted in clinically and statistically insignificant tissue rebound [42]. This aligned with another clinical study by Carneiro et. al [44]. Therefore, to prevent coronal migration in these cases, gingival thinning was performed for phenotype conversion, a 3‐mm distance between the CEJ and alveolar crest was maintained, and the flap was sutured 3 mm from the osseous crest.

During the restorative phase, the literature commonly advocates tooth preparation followed by porcelain‐fused‐to‐metal crowns, zirconia crowns, or lithium disilicate porcelain veneers after crown lengthening [22–24]. However, these restorative options are inherently invasive. The present case series highlights a minimally invasive restorative approach using a nanocomposite material. Beautifil XSL (Shofu) is a bioactive nano‐hybrid composite containing nano‐sized multifunctional surface pre‐reacted glass (S‐PRG) fillers that exhibit moisture tolerance before incorporation into the resin matrix. This giomer material exhibits a trilaminar structure composed of an outer surface‐modified layer, an intermediate glass‐ionomer phase, and an inner multifunctional glass core. The trilaminar configuration of the S‐PRG filler forms a stable glass‐ionomer structure that enables ion release and recharge while protecting the glass core from moisture, thereby enhancing the longevity of the material. In addition to acting as a local reservoir of fluoride ions for uptake by enamel and dentin, it also helps inhibit the development of recurrent caries. S‐PRG filler particles are produced by reacting fluoride‐containing acid‐reactive glass with polyalkenoic acid in water before incorporation into the resin matrix. This process differs from that of compomers, where the reaction between dehydrated polyalkenoic acid and glass occurs only after water absorption by the restorative material. S‐PRG fillers uniquely release multiple ions, including fluoride, sodium, strontium, aluminum, silicate, and borate. Silicate and fluoride ions are known to promote remineralization of the dentin matrix, whereas fluoride and strontium interact with hydroxyapatite to form fluorapatite and strontium‐apatite, respectively, thereby increasing tooth resistance. In addition to its bioactivity, the material provides lifelike esthetics, and the combination of favorable handling characteristics, optical properties, and higher microtensile bond strength. Improved bond durability at the resin–dentin interface may be attributed to strengthened dentin resulting from fluoride ion uptake, as well as the retention of relatively insoluble 4‐acryloxyethyl‐trimellitic acid calcium salts formed around residual apatite crystallites within the hybrid layer of self‐etch adhesives [46]. In addition, the S‐PRG filler releases five ions—sodium, strontium, aluminum, silicate, and borate—conferring bioactive properties. Upon exposure to lactic acid, these ions contribute to acid‐neutralizing effects [47].

Several studies have reported on techniques for midline diastema and maxillary anterior space closure. Various approaches have been described, including the use of a putty index, posterior sectional matrix, freehand composite layering, and a double putty index technique [48–51]. However, most of these reports do not specifically address the rationale for restorative approaches selection. A randomized clinical trial conducted in 2023 compared two matrix systems—Bioclear matrix and conventional celluloid matrix with TorVM sectional matrix for the restoration of black triangles [52]. The study reported that both protocols could provide superior esthetic outcomes, good marginal adaptation, suitable biological properties, and adequate survival time. Nevertheless, the clinical outcome largely depended on the operator′s skill. The authors recommended that clinicians ensure proper cervical strip closure in the apical direction when using the celluloid matrix technique along with sectional matrix and select the appropriate matrix size using a gauge when employing the Bioclear system. These measures help achieve an optimal emergence profile, appropriate contact point and contour, and minimize the risk of overhanging restorations.

To ensure functional stability, a canine guidance occlusal scheme was established for both cases. This restorative approach is supported by the fact that composite resin veneers exhibit greater deformation than more rigid alternatives, indicating a superior capacity for stress absorption [31]. Such a property is highly advantageous for canine guidance, where the teeth must withstand significant lateral forces during excursive movements [31]. By leveraging the flexible nature of the bioactive nano‐hybrid composite, these functional forces are dissipated more effectively, thereby reducing the risk of catastrophic fracture often associated with brittle ceramic restorations [31]. This clinical strategy is further validated by a 2‐year longitudinal study by Utsumi et al. on the reconstruction of canine guidance [53]. Their research, which utilized IOS data, demonstrated that although minor wear and marginal discoloration may occur, they remain clinically acceptable and do not compromise the overall success of the restorations [54].

Evidence‐based comparisons suggest that although indirect ceramic veneers are often preferred for their superior color stability and long‐term fracture resistance, direct composites are highly effective in clinical scenarios where the maximum preservation of tooth structure is a priority [28, 30]. Studies show that ceramic restorations typically maintain survival rates above 90% over 10 years, whereas direct composite buildups exhibit a respectable 5‐year survival rate of approximately 84.6% [28, 30]. Despite a higher susceptibility to surface roughness and marginal staining over time compared with ceramics, composites can achieve a 100% functional survival rate due to their unique ease of repairability [28, 30]. Minor chipping, identified as the most common complication for composites, can be resolved cost‐effectively without re‐entering the cycle of repeated invasive restoration, making them an ideal choice for adolescents and young adults with intact enamel [28, 29]. Consequently, although ceramics remain a gold standard for long‐term durability, modern nano‐hybrid composites provide a statistically comparable and conservative treatment alternative with high objective esthetic scores. Previous studies have reported favorable longevity for nano‐hybrid composite Class IV restorations, with a 4‐year survival rate of approximately 94.6%, minimal annual failure rates, fracture being the most common mode of failure, and satisfactory optical properties [27].

The limitations of this case series include the 24‐month (Case 1) and 20‐month (Case 2) follow‐up duration, which represents a relatively brief window for assessing the stability of both periodontal and restorative outcomes such as potential tissue rebound, the color stability of the nanocomposite material and its complications like fracture/chipping. This study also shows the inability of composite restorations to correct midline discrepancies, as observed in the first case. Future studies with long‐term observation periods are necessary to confirm the long‐term maintenance of the esthetic zenith and the structural integrity of the composite emergence profile.

## 4. Conclusion

Esthetic crown lengthening followed by direct composite veneering represents a predictable and minimally invasive treatment modality for the management of gummy smile associated with APE Type I and AAE. Accurate diagnosis and meticulous preservation of the supracrestal tissue attachment are essential for achieving marginal gingival stability and favorable pink esthetic outcomes. The use of direct composite restorations further enhances white esthetic parameters while preserving sound tooth structure. Additional advantages of this approach include reduced procedural invasiveness, shorter treatment duration, and lower overall cost, making it a conservative and clinically viable alternative to conventional prosthetic restorative options.

## Funding

No funding was received for this manuscript.

## Consent

Written informed consent was obtained from the patients for the publication of the case series, accompanying clinical photographs, and treatment details.

## Conflicts of Interest

The authors declare no conflicts of interest.

## Data Availability

Data sharing is not applicable to this article as no datasets were generated or analyzed during the current study.
